# Rapid Phenotypic Antibiotic Susceptibility Profiling of Clinical *Escherichia coli* and *Klebsiella pneumoniae* Blood Cultures

**DOI:** 10.3390/antibiotics13030231

**Published:** 2024-02-29

**Authors:** Idan Hefetz, Rita Bardenstein, Shahar Rotem, Galia Zaide, Gal Bilinsky, Ohad Shifman, Oren Zimhony, Ronit Aloni-Grinstein

**Affiliations:** 1 Department of Biochemistry and Molecular Genetics, Israel Institute for Biological Research, Ness Ziona 7410001, Israel; idanh@iibr.gov.il (I.H.); shaharr@iibr.gov.il (S.R.); galiaz@iibr.gov.il (G.Z.); galb@iibr.gov.il (G.B.); ohads@iibr.gov.il (O.S.); 2 Infectious Diseases Unit, Kaplan Medical Center Faculty of Medicine, Hebrew University of Jerusalem, Rehovot 7661041, Israel; rita_b@clalit.org.il

**Keywords:** bloodstream infection, *Escherichia coli*, *Klebsiella pneumoniae*, MAPt, antibiotic susceptibility

## Abstract

Bloodstream infections (BSI) are defined by the presence of viable bacteria or fungi, accompanied by systemic signs of infection. Choosing empirical therapy based solely on patient risk factors and prior antibiotic susceptibility test (AST) may lead to either ineffective treatment or unnecessarily broad-spectrum antibiotic exposure. In general, Clinical & Laboratory Standards Institute guideline-approved ASTs have a turnaround time of 48–72 h from sample to answer, a period that may result in a critical delay in the appropriate selection of therapy. Therefore, reducing the time required for AST is highly advantageous. We have previously shown that our novel rapid AST method, MAPt (Micro-Agar-PCR-test), accurately identifies susceptibility profiles for spiked bioterrorism agents like *Bacillus anthracis*, *Yersinia pestis* and *Francisella tularensis* directly from whole-blood and blood culture samples, even at low bacterial levels (500 CFU/mL). This study evaluated the performance of MAPt on routine bloodstream infection (BSI), focusing on *Escherichia coli* and *Klebsiella pneumoniae* isolates from clinical cultures, including resistant strains to some of the six tested antibiotics. Notably, MAPt yielded results exceeding 95% agreement with the standard hospital method within a significantly shorter timeframe of 6 h. These findings suggest significant potential for MAPt as a rapid and reliable BSI management tool.

## 1. Introduction

Bloodstream infections (BSIs) are defined by positive blood cultures in a patient with systemic signs of infection. Generally, BSIs represent 40% of cases of sepsis and septic shock and approximately 20% of intensive care unit (ICU)-acquired cases [1]. ICU BSIs carry a greater risk of mortality, with a 40% increase in the risk of 30-day death, particularly when prompt adequate antibiotic therapy is delayed [2]. In cases of bacteremia, the mortality rate doubles when there is a 24 h delay in the administration of appropriate antimicrobials [3]. A recent study by the National Institute for Antibiotic Resistance and Infection Control, Ministry of Health, Tel Aviv, Israel revealed that nearly 50% of adult hospital-onset BSI and multidrug-resistant (MDR) BSI patients—with infection caused by eight sentinel bacteria: *Escherichia coli*, *Klebsiella pneumoniae*, *Pseudomonas aeruginosa*, *Acinetobacter baumannii*, *Streptococcus pneumoniae*, *Staphylococcus aureus*, *Enterococcus faecalis* and *Enterococcus faecium*—died within 1 year. Mortality increased with age, but in all age groups, survival continued to decline for a year after the acute event [4,5]. Others have also demonstrated a strong association between nosocomial BSIs and increased risk of death, persisting up to 1 year after infection [6].

*Escherichia coli (E. coli)*, an *Enterobacteriaceae*, is the most common cause of bacteremia in high-income countries [7]. Emerging multidrug-resistant *E. coli* strains pose a greater treatment challenge and confer a higher risk of bacteremia and death [8,9,10]. In Israel, the annual incidence and case fatality rates of *E. coli* BSI are about twice as high compared to previous reports from other high-income countries, largely due to high resistance levels [4]. 

Excessive antibiotic use across various classes is a risk factor for the emergence of resistance. The key resistance mechanisms involve the production of β-lactamases (particularly extended-spectrum β-lactamases, termed ESBLs), cephamycinases and carbapenemases [11]. ESBLs can be inhibited by β-lactamase inhibitors such as clavulanic acid, sulbactam, taxobactam, avibactam and relebactam [12,13,14]. ESBLs pose a significant threat and often are associated with resistance to fluoroquinolones (e.g., ciprofloxacin), and aminoglycosides (e.g., gentamicin) [11,15,16]. Thus, shortening the time of antibiotic susceptibility testing (AST) is crucial.

Empiric therapy based on patient risk factors and traditional culture-based antibiograms may fail to predict or reveal susceptibility within clinically relevant timeframes that may enable timely adjustment. Unfortunately, routine ASTs using methods like disk diffusion and broth microdilution typically require 48–72 h for culture growth, identification and susceptibility profiling of the pathogen, significantly exceeding the optimal window for initial therapeutic decisions. This delay potentially leads to treatment failure and increased mortality. To overcome a potential delay, empiric usage of broad-spectrum regimens is employed, which may further contribute to antibiotic resistance. Thus, shortening the time of antibiotic susceptibility testing (AST) is highly desirable.

With advancing knowledge of resistance mechanisms, genetic ASTs have emerged, testing for gene presence/absence or resistance mutations as markers for resistance. While these methods offer faster results, phenotypic validation remains necessary to ensure the genomic marker’s predictive accuracy, as the effects of various mutations and resistance genes can differ. Additionally, the dynamic nature of resistance emergence raises concerns about unforeseen resistance due to novel polymorphisms of genes [17]. A rapid, phenotypic AST method independent of prior knowledge about resistance mechanisms would be highly advantageous. Ideally, it should be simple and cheap to perform, easy to apply to various bacteria/antibiotic combinations, reliable and provide accurate results within a significantly shorter timeframe.

We have developed MAPt (Micro-Agar-PCR-test), a rapid phenotypic AST method that allows one to address bioterror agent-contaminated environmental samples [18] as well as whole-blood and blood culture samples [19]. MAPt is based on the direct application of a sample onto solid agar that has been embedded with different concentrations of the tested antibiotic. Following a short incubation, bacterial growth is examined by qPCR. Using agar medium, which better supports the growth of bacteria at low concentrations, together with the application of qPCR, which provides sensitivity and specificity, allows for minimal inhibitory concentration (MIC) determination to a wide range of bacterial concentrations, ranging from ~5 × 10^2^ cfu/mL up to 10^8^ cfu/mL. In this study, we describe the application of MAPt to clinical blood culture samples harboring *E. coli* or *K. pneumoniae*, some with antibiotic resistance to the tested antibiotics. Our results demonstrate a good correlation between MAPt-derived susceptibility/resistance categories and those obtained by standard methods at Kaplan Medical Center, Israel (KMC), within a significantly shorter timeframe.

## 2. Results

### 2.1. MAPt vs. Kirby–Bauer Disc Diffusion

Sixty-three clinical isolates of *E. coli* (forty-nine isolates) and *K. pneumonia* (fourteen isolates) were subjected to susceptibility categorization by MAPt at the Israel Institute for Biological Research (IIBR) and the results were compared to the ones obtained by Kirby–Bauer disc diffusion using zone diameter and MIC interpretive standards at KMC. We chose six bactericidal antimicrobials most commonly used in the clinic for treating *E. coli* and *Klebsiella pneumonia* infections, mostly originating from urinary tract infections that eventually result in bacteremia.

Table 1 summarizes the comparison between the different AST techniques and Table S1 provides the raw data.

Comparing MAPt to disc diffusion revealed that the numbers of major and minor errors for all six agents were low, meeting FDA requirements. However, MAPt did not meet the FDA requirement of <1.5% very major error compared to microdilution for ceftriaxone, amikacin and ceftazidime.

Despite this, all six agents had categorical agreement above 95%. Overall, all six agents complied with FDA requirements for very major, major and minor errors (Table 1 last line).

Five out of sixty-three samples exhibited category discrepancies with ceftriaxone and ceftazidime. As the antibiotic susceptibility test to these agents is performed in KMC by positive double-disk synergy test, using ceftriaxone or ceftazidime and amoxicillin–clavulanate (AMC) disks, we examined whether the addition of clavulanate to the MAPt test setting may account for the difference between the two tests. Therefore, we prepared MAPt plates containing ceftriaxone without or with potassium clavulanate to a final concentration of 4 μg/mL and examined the difference between the MIC obtained by these two types of plates. For the disc diffusion assay, the test is considered positive when a decreased susceptibility to ceftriaxone is combined with a clear-cut enhancement of the inhibition zone of cefotaxime in front of the clavulanate-containing disk. Likewise, Etest ESBL strips are two-sided strips that contain a gradient of ceftazidime on one end and ceftazidime plus clavulanate on the other end. A positive test for an ESBL is indicated by a three-dilution reduction in the ceftazidime MIC in the presence of clavulanic acid. This test was shown to be more sensitive than the double-disk approximation test in detecting ESBLs in clinical isolates [20].

Comparison of MAPt MIC values for ceftriaxone with and without clavulanate revealed a greater-than-three-dilution reduction for three of the five tested samples (#36, #37, #55), classifying them as resistant. However, adding clavulanate did not resolve the discrepancies for samples #1 and #7. Therefore, we performed additional susceptibility testing: microdilution, Etest and a 24 h micro agar test with visual examination for the latter two. Sample #1 tested sensitive by MAPt, Etest and micro agar test, but resistant by microdilution and the double-disk test. Sample #27 tested sensitive by MAPt and micro agar but resistant by Etest, microdilution and the double-disk test. The reason for these discrepancies remains unclear.

### 2.2. MAPt vs. Microdilution

MAPt was compared to microdilution to define essential categories. The susceptibilities of the two quality control strains for anaerobes were tested at the time of production of broth microdilution and MAPt trays and each time a batch of clinical strains were examined. All quality control strains were tested at least three times during the clinical isolate test period. All quality control strains were within acceptable ranges for all antimicrobials tested and were highly reproducible. Table 2 summarizes the very major, major and minor error rates, as well as the categorical and essential agreements between MAPt and microdilution for 63 clinical isolates of *E. coli* (49 isolates) and *K. pneumoniae* (14 isolates) tested against six commonly prescribed antimicrobial agents. Raw data are available in Table S2.

The numbers of major and minor errors for all six agents were low, meeting FDA requirements. However, MAPt did not meet the FDA requirement of <1.5% very major error compared to microdilution for ceftriaxone, amikacin and ceftazidime.

Despite this, all six agents had categorical agreement equal to or above 90%, and all but ceftazidime had an essential agreement exceeding 90% compared to microdilution. Overall, all six agents complied with FDA requirements for very major, major and minor errors, as well as categorical and essential agreements (Table 2 last line).

### 2.3. Genome Assembly and Resistance Genes Identification Results

A genomic approach was applied to reconcile discrepancies observed between MAPt and disc diffusion assays. Note that the genomic approach typically predicts family-level resistance profiles rather than susceptibility to a specific drug. *E. coli* strains E27, E55 and E63 exhibited ceftriaxone and ceftazidime resistance by both tests, while E7 tested for ceftriaxone susceptible by MAPt and resistant by disc diffusion. E42, sensitive by both methods, served as a reference for a sensitive profile. Similarly, disc diffusion indicated amikacin resistance in E27 and E63 while the MAPt test resulted in a susceptible phenotype for both isolates. E7, E42 and E55 tested amikacin-sensitive by both methods (Table S2). A computational analysis of the previously sequenced isolates was carried out to explore their “Resistome”. To this end, the sequencing data of each isolate were assembled, resulting in good quality control metrics (72–113 contigs, N50 147–295 kbp). Resequencing against assembled contigs achieved adequate coverage (55–120×). The assembled genomes were then subjected to a computational antimicrobial resistance (AMR) analysis using the CARD Resistance Gene Identifier (RGI) tool https://card.mcmaster.ca/analyze/rgi (accessed on 8 January 2023). The analysis revealed the presence of various resistance genes and mutations, potentially conferring resistance to several drug classes.

**Figure 1:**
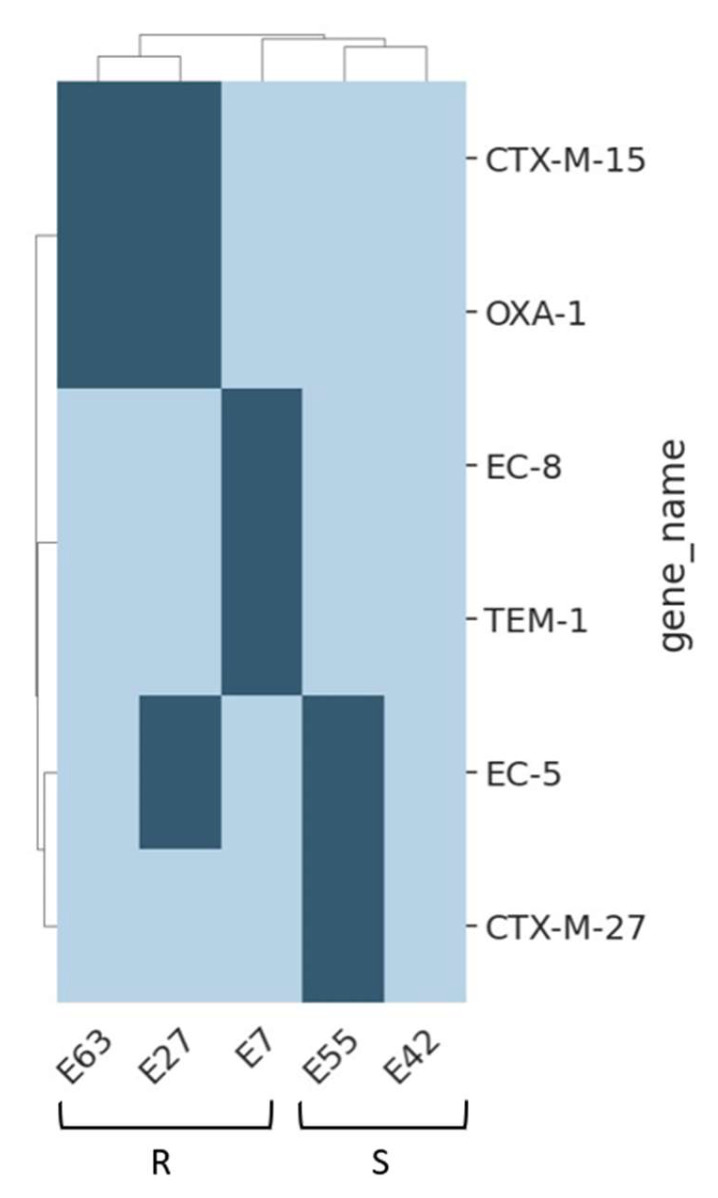
Highlighting the absence of cephalosporin resistance genes in E42 compared to other resistant *E. coli* isolates. The map presents a clustered presence/absence (dark/light) table for cephalosporin-associated resistant genes (rows) found in five *Escherichia coli* samples (columns). R-resistant strains, S-sensitive strains as designated by the disc-diffusion test. Items on both axes are clustered. Genes that exist in all samples are not shown.

**Figure 2:**
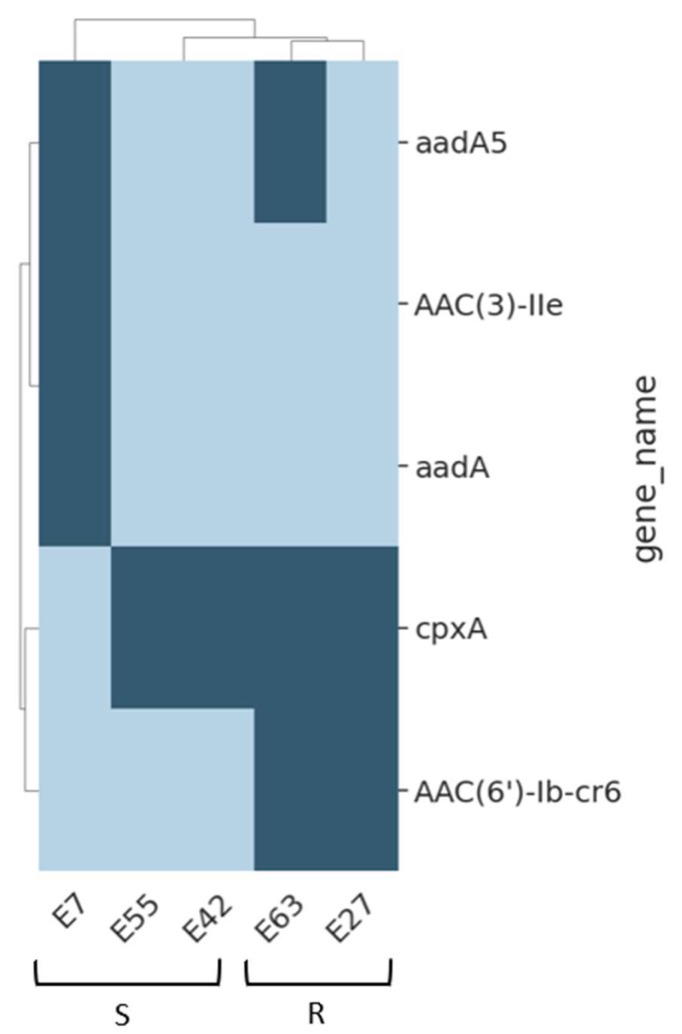
Highlighting the presence of aminoglycoside resistance gene in E27 and E63 compared to other sensitive *E. coli* isolates. The map presents a clustered presence/absence (dark/light) table for aminoglycoside-associated resistant genes (rows) found in five *E. coli* samples (columns). Items on both axes are clustered. R-resistant strains, S-sensitive strains as designated by the disc-diffusion test. Genes that exist in all samples are not shown.

To elucidate potential associations between genes and specific drug resistance phenotypes, we employed hierarchical clustering. Analyses were conducted for cephalosporins and aminoglycosides, iteratively grouping genes based on their presence/absence across isolates. The resulting clustered heat maps (Figure 1 and Figure 2) visually depict these gene clusters. Genes appearing in all samples are not shown for clarity (the full list is provided in Supplementary Table S3). AST for the *E. coli* isolates revealed a resistance pattern to the cephalosporin drugs ceftriaxone and ceftazidime for all samples but E42 thus coinciding with the observation that E7 is resistant to ceftriaxone, as determined by the disc diffusion test (Figure 1A and Table S2). The resistance to ceftriaxone is likely attributed to the presence of the CTX-M-15, OXA-1, TEM-1, EC-8, EC-5 and CTX-M-27 found only among the phenotypically resistant isolates (Figure 1). These genes encode enzymes that inactivate cephalosporin antibiotics, explaining the observed resistance. Notably, the E42 isolate lacks these genes, potentially explaining its susceptibility to cephalosporins. CTX-M-15 for example, an extended-spectrum β-lactamase (ESBL), exhibits high efficiency against oxyimino-cephalosporins like cefotaxime and ceftazidime, leading to significant resistance [11,21]. Likewise, CTX-M-27 resembles CTX-M-15 in its substrate profile and resistance spectrum, targeting oxyimino-cephalosporins with high efficiency [22]. Their prevalence, particularly in Enterobacteriaceae like *Escherichia coli* and *K. pneumoniae*, makes them a major public health concern. The oxa-1 gene encodes a class D β-lactamase, a type of enzyme that confers resistance to a broad spectrum of β-lactam antibiotics, including cephalosporins, penicillins, and some carbapenems. This β-lactamase has high catalytic efficiency, rapidly hydrolyzing β-lactam. Although oxyimino-cephalosporins, such as ceftazidime and cefotaxime, are poor substrates for TEM-1, the use of these antibiotics primed the evolution of TEM variants that significantly augmented the hydrolysis of ceftazidime and cefotaxime [23].

BlaEC-8 was shown to mediate resistance to cephalosporins including third- (ceftazidime) and fourth-generation (cefepime) cephalosporins [24,25].

Similarly, resistance to the aminoglycoside amikacin was observed in the E27 and E63 isolates but not in E7, E42, or E55 (Table S2). This discrepancy may be due to the presence of the aac(6′)-lb-cr6 gene in the resistant isolates (Figure 2). The aac(6′)-lb-cr6 gene encodes an aminoglycoside 6’-N-acetyltransferase (AAC(6′)-Ib-Cr6), which catalyzes the acetylation of the 6’-amino group of aminoglycosides, effectively disrupting their binding to the bacterial ribosome and inhibiting protein synthesis, conferring broad-spectrum resistance to a wide range of aminoglycoside antibiotics, including amikacin, gentamicin, kanamycin and tobramycin, in diverse bacterial species, including *Enterobacteriaceae* and *Pseudomonas aeruginosa* [26]. Subsequent variants like aac(6’)-Ib-Cr4 and aac(6’)-Ib-Cr6 have acquired an additional function. These variants can acetylate a specific nucleotide within the quinolone-resistant DNA gyrase enzyme, the primary target of fluoroquinolones, effectively disabling its function and providing broad-spectrum resistance. Indeed, both E27 and E63 isolates possess resistance to ciprofloxacin as well (Table S2).

## 3. Discussion

The present study compared the performance of MAPt with Kirby–Bauer disc diffusion and microdilution broth for the susceptibility testing of six commonly used antibiotics against Gram-negative bacteria, specifically *E. coli* and *K. pneumoniae* isolated from a clinical setting. Overall, the results suggest that MAPt is a promising alternative to traditional antimicrobial susceptibility tests, exhibiting several advantages but also harboring some limitations.

Comparing MAPt with the disc diffusion assay has revealed an overall high categorical agreement (97.8%). For all antibiotics, MAPt demonstrated excellent agreement with the disc diffusion assay exceeding the FDA requirement for >90%. This indicates that MAPt accurately categorizes isolates as susceptible or resistant in most cases. MAPt observed high categorical and essential agreement with the microdilution test, conducted at IIBR. For five out of six antibiotics, MAPt demonstrated excellent agreement with the gold standard microdilution method, exceeding 90% for both categorical and essential agreement. This indicates that MAPt accurately categorizes isolates as susceptible, intermediate, or resistant in most cases and that the MIC value obtained by both methods is similar. This study found that MAPt complied with FDA requirements showing low error rates for very major, major and minor errors, indicating high reproducibility and reliability. Moreover, MAPt offers a faster turnaround time compared to traditional methods like microdilution, potentially allowing for quicker antibiotic selection and improved patient outcomes. 

Further studies with larger sample sizes for all six antibiotics are warranted to fully assess the comparative accuracy of MAPt to both methods. Whole-genome sequencing and resistance gene identification tools allowed for the identification of specific genes likely involved in the observed resistance phenotypes observed by the disc diffusion disk but not by MAPt. For example, the presence of TEM-1 and EC-8 in E7 isolate favors the observed resistant phenotype scored by the disc diffusion assay compared to the sensitive phenotype obtained by MAPt. Likewise, the presence of aac(6′)-lb-cr6 coincides with the resistant phenotype of E27 and E63 isolates as determined only by the disc diffusion assay but not by MAPt. It should be noted, however, that the presence of a resistant gene is not a determinant factor for a resistant phenotype. Yet, the presence of several resistant genes may point to a more conclusive determination. 

To conclude, the emergence of multidrug-resistant bacteria necessitates the development and integration of rapid and reliable bacterial identification methods such as the T2MR, which provides identification within 6 h [27], and most importantly phenotypic AST methods like MAPt, which are crucial for swift determination of bacterial susceptibility. While further research is needed to address potential limitations and optimize its implementation, MAPt’s speed, accuracy and broad-spectrum coverage suggest its significant potential as a valuable tool for optimizing antimicrobial therapy, improving patient outcomes and curbing the spread of antibiotic resistance. 

## 4. Materials and Methods

### 4.1. Bacterial Strains

All *E. coli* and *K. pneumonia* strains were obtained from blood cultures that were taken at KMC as part of the diagnostic workup of patients with sepsis as determined by discretion of the treating physicians. The BACTEC blood cultures were held at 37 °C before shipment to IIBR. Two milliliters of samples from blood culture bottles of identified *E.coli* and *K. pneumonia* were transferred to the current study for a blinded MAPt test at the IIBR institute according to a protocol approved by the KMC Helsinki committee (KMC 128-21). 

### 4.2. Media and Growth Conditions

Bacteria were grown on Brain Heart Infusion agar (BHI-A; BD Difco 241830, Le Pont de Claix, France) plates at 37 °C. Determination of bacteria load (CFU/mL) from blood culture was calculated by plating 100 μL of serial 10-fold dilutions in phosphate-buffered saline (PBS, Biological Industries, Beit HaEmek, Israel) on Brain Heart Infusion agar (BHI-A; BD Difco 241830) plates at 37 °C.

### 4.3. Antibiotic-Supplemented

The following antibiotic stock solutions were prepared: 4 µg/mL ceftriaxone (Sigma Aldrich Cat. C5793, St. Louis, MO, USA), 4 µg/mL ertapenem (Sigma Aldrich Cat. SML1238, St. Louis, MO, USA), 4 µg/mL meropenem (Sigma Aldrich Cat. M2574 St. Louis, MO, USA), 64 µg/mL amikacin (Sigma Aldrich Cat. A1774, St. Louis, MO, USA), 16 µg/mL ceftazidime (Sigma Aldrich Cat. CDS020667, St. Louis, MO, US.A) and ciprofloxacin (CIPRO-TEVA^®^ 2 mg/mL, Demos. A. Pharmaceutical Industry, Thermi, Greece). Twofold serial dilutions were made from these stock solutions by adding 25 µL antibiotic to 25 µL of double distilled sterilized water.

### 4.4. Preparation of MAPt Plates

MAPt plates were prepared using Mueller-Hinton Agar (MHA; Difco 0252-17-6) according to the manufacturer’s instructions, followed by autoclaving at 120 °C for 20 min and chilling of the agar in a water bath to 50 °C. A stock solution of the tested antibiotic was diluted to the concentration that was defined as a resistance breakpoint, according to the CLSI standard M07 (CLSI, 2022) recommendation. Antibiotic serial dilutions were made as follows: five parts of molted agar (125 μL) was added on top of the 25 μL diluted antibiotics in a 96-well plate, where agar-containing wells with no antibiotics served as growth control. In some cases, MAPt plates containing ceftriaxone were also supplemented with a final concentration of 4 μg/well of potassium clavulanate (Sigma-33454). MAPt plates may be stored for 2 months at 4 °C or up to a year at −70 °C [28].

### 4.5. Samples Description, Identification and Antibiotic Susceptibility Determination by Kirby–Bauer Disc Diffusion Assay

Blood culture bottles, either BACTEC plus aerobic/F culture vials (BD 442023) or BACTEC Lytic/10 anaerobic/F culture vials (BD 442021), were incubated at 37 °C, typically 1–5 days in the BD BACTEC Fx automated system until growth was demonstrated. *E. coli* and *K. pneumonia* strains were identified as such from bactec Dickinson blood culture bottles by either CHROMagar™ Orientation Chromagar Hylab (Rehovot, Israel), MALDI-TOFF Mass Spectrometry Bruker (Shimaduz Europa GmdH, Kyoto Japan), or Vitek 2 ^®^Healtcare BioMerieux (Durham, NC, USA). Susceptibility to an antimicrobial agent was determined by Kirby–Bauer disc diffusion using zone diameter and MIC interpretive standards, or Vitek 2 ^®^Healtcare BioMerieux. All isolates were subject to double disk synergy test (DDST). All these tests were according to CLSI guidelines [29]. Susceptibility was determined by disk diffusion and vitek-2 system according to CLSI guidelines. The BACTEC blood cultures were held at 37 °C, before shipment to IIBR institute. Two milliliters of samples from blood culture bottles of identified *E. coli* and *K. pneumonia* were transferred to the current study for a blinded MAPt test at the IIBR institute according to a protocol approved by the KMC Helsinki committee (128-21). The MAPt was blinded for susceptibility while the identification of either *E. coli* or *K. pneumonia* was reported to allow for appropriate primer usage.

### 4.6. MAPt Assay

Ten microliters of the tested blood-infected culture were plated in 96-well MAPt plates containing different concentrations of the tested antibiotics (each sample was tested at duplicate repeats). MAPt plates were incubated for four hours at 37 °C. Following the incubation period, the bacteria were extracted from the MAPt plates by adding 150 μL of PBS and shaking the plate for two minutes at 1000 rpm in a TALBOYS professional incubating microplate shaker. 

A hundred microliters of the recovered bacteria in the PBS buffer were added to 100 μL of Triton buffer (20% Triton-X-100 in TE, Sigma, Rehovot, Israel) in a PCR plate. Samples were heated for 30 min at 100 °C and a sample of 5 μL bacterial extract was transferred to a 96-well PCR plate for qPCR analysis using the 7500 Real-Time PCR system (Applied Biosystems, Foster City, CA, USA).

### 4.7. qPCR Reaction

The qPCR reactions were performed in 30 μL volume containing 2.3 μL of 20 mg/mL bovine serum albumin (BSA; Sigma A2153, Rehovot, Israel), 15.05 μL SensiFAST Probe Lo-ROX Mix (Bioline BIO84005, Cincinnati, OH, USA), 3.05 μL forward primer (5 pmol/μL), 3.05 μL reverse primer (5 pmol/μL) and 1.55 μL TaqMan Probe. Primers and probes for *E. coli* and *K. pneumonia* were ordered from Integrated DNA Technologies, according to [30]. The estimated price for an assay for one antibiotic is approximately 8–10 dollars. 

### 4.8. qPCR Program

The following steps were taken to perform qPCR test: 94 °C for 10 s followed by 40 cycles of denaturation at 94 °C for 10 s, annealing at 55 °C for 30 s and extension at 72 °C for 30 s. 

### 4.9. Analysis

Real-time qPCR was employed to estimate bacterial load using cycle threshold (Ct) values generated by Sequence Detection Software (SDS v1.4, Applied Biosystems). Relative growth differences (FC) between antibiotic-treated and untreated control samples were determined by FC = 2^−ΔCt^, where ΔCt represents the difference in Ct values between treated and control samples. MIC was defined as the lowest antibiotic concentration resulting in a ΔCt ≥ 3.3, indicative of at least a 10-fold reduction in growth compared to the control. This definition aligns with the absence of visible growth observed in standard antibiotic susceptibility testing (AST) methods. Accordingly, MIC values were calculated from the Ct values through a Microsoft Excel spreadsheet (Supplementary File S1).

All MICs were interpreted using CLSI clinical breakpoints. The comparison of MAPt to the reference methods was performed using categorical and essential agreement, a method commonly used to compare different antimicrobial susceptibility testing (AST) methods. Categorical agreement is based on interpretive breakpoints of sensitive (S), intermediate (I) and resistant (R). A very major error is defined as the reference result being resistant and the test result being susceptible. A major error is defined as the reference result being susceptible and the test result being resistant. A minor error is defined as the reference result being resistant or susceptible and the test result being intermediate, or the reference result being intermediate and the test result being susceptible or resistant. Essential agreement is based on the number of MICs plus or minus one doubling dilution of the reference MIC. The FDA requires >90% essential and category agreement, <1.5% very major errors, <3% major errors and <10% minor errors.

### 4.10. MIC Determination by Broth Microdilution

Standard broth microdilution was performed according to the CLSI guidelines [29].

### 4.11. Computational Methods

Whole-genome, paired-end sequencing was conducted by SeqCenter Pittsburgh (Pittsburgh, PA, USA), FastQC with default settings was used for quality control of the data. Trimming and removal of low-quality reads were performed using FAQCS with default settings. Assembly for each sample was generated using IDBA_UD assembler with default parameters and validated by resequencing of the FASTQ files against the assembled contigs using Bowtie 2. Prediction of AMR (antimicrobial resistance) genes and elements was carried out using the CARD Resistance Gene Identifier (RGI) tool version 6.0.2 with CARD database version 3.2.7 (https://card.mcmaster.ca/analyze/rgi, accessed on 8 January 2023). Results were divided by drug classes (e.g., fluoroquinolones, aminoglycosides, etc.) and clustered heatmaps were generated by applying the Seaborn “Clustermap” module in a Python script. (https://seaborn.pydata.org/generated/seaborn.clustermap.html, accessed on 9 July 2023).

## Figures and Tables

**Table 1:** Performance of MAPt versus disc diffusion.

Antimicrobial Agent	No. of Strains in Each Resistance Category			No. (%) of Errors						
	S	I	R	Minor		Major		Very Major		% Agreement (CA *)
Ceftriaxone	33	0	30	0	(0)	0	(0)	2	(3)	97
Ertapenem	61	0	2	0	(0)	0	(0)	0	(0)	100
Meropenem	60	0	2	0	(0)	2	(3)	0	(0)	97
Amikacin	60	0	3	0	(0)	1	(1.5)	2	(3)	95.5
Ciprofloxacin	26	0	16	0	(0)	0	(0)	0	(0)	100
Ceftazidime	24	0	18	0	(0)	0	(0)	1	(2.5)	97.5
Total				0	(0)	3	(1)	5	(1.2)	97.8

* CA: categorical agreement defined by comparison to disc diffusion.

**Table 2:** The number of categorical errors for MAPt compared to microdilution.

Antimicrobial Agent	No. of Strains in Each Resistance Category			No. (%) of Errors						
	S	I	R	Minor		Major		Very Major		% Agreement (CA/EA *)
Ceftriaxone	33	0	30	0	(0)	0	(0)	2	(3)	97/95
Ertapenem	61	0	2	1	(1.5)	0	(0)	0	(0)	98.5/95
Meropenem	60	0	2	4	(6)	2	(3)	0	(0)	91/94
Amikacin	59	0	2	1	(1.5)	1	(1.5)	2	(3)	94/93
Ciprofloxacin	26	0	14	0	(0)	0	(0)	0	(0)	100/95
Ceftazidime	24	0	16	2	(5)	1	(2.5)	1	(2.5)	90/85
Total				8	(2)	4	(1)	5	(1.2)	95.8/93

* CA: categorical agreement defined by comparison to microdilution; EA: essential agreement defined by comparison to microdilution.

## Data Availability

The data presented in this study are available upon request from the corresponding authors.

## Supplementary Materials

The following supporting information can be downloaded at: https://www.mdpi.com/article/10.3390/antibiotics13030231/s1, Table S1: MIC values obtained by MAPt versus MBD. Marked in yellow are discrepancies between MIC values obtained by the two methods. Table S2: Susceptibility categories obtained by MAPt versus disk diffusion. Marked in yellow are discrepancies between susceptibility categories obtained by the two methods, in light blue resistant strains and green susceptible strains, according to the tested antibiotic. Table S3: List of resistant genes that appear both in phenotypically tested resistant and susceptible strains and were omitted from Figure 1A,B for clarity. File S1: MAPt raw data.
