# Supplementary material for: Rapid Phenotypic Antibiotic Susceptibility Profiling of Clinical Escherichia coli and Klebsiella pneumoniae Blood Cultures
Source: Antibiotics (Basel). 2024 Feb 29;13(3):231. doi: 10.3390/antibiotics13030231 (PMC10967342; doi:10.3390/antibiotics13030231)
Supplement: Supplementary file 1 [file antibiotics-13-00231-s001.zip › Supplementary Table S3.pdf]

Supplementary Table S3:

For figure 1(A-B), genes that appear in all samples are not shown. The list of those resistance genes is provided here (names as they appear in the CARD database):

Escherichia coli, Cephalosporin
'Escherichia coli soxR with mutation conferring antibiotic resistance', 'Escherichia coli AcrAB-TolC with AcrR mutation conferring resistance to ciprofloxacin, tetracycline, and ceftazidime', 'Escherichia coli acrA', 'marA', 'Escherichia coli soxS with mutation conferring antibiotic resistance','Klebsiella pneumoniae KpnF', 'acrB', 'Klebsiella pneumoniae KpnE', 'H-NS','Haemophilus influenzae PBP3 conferring resistance to beta-lactam antibiotics','TolC', 'AcrF', 'AcrS', 'AcrE','Escherichia coli AcrAB-TolC with MarR mutations conferring resistance to ciprofloxacin and tetracycline'

Escherichia coli, Aminoglycosides
'acrD', 'baeS', 'baeR', 'kdpE', 'Klebsiella pneumoniae KpnF', 'TolC','Klebsiella pneumoniae KpnE'
