# Supplementary material for: Rapid Phenotypic Antibiotic Susceptibility Profiling of Clinical Escherichia coli and Klebsiella pneumoniae Blood Cultures
Source: Antibiotics (Basel). 2024 Feb 29;13(3):231. doi: 10.3390/antibiotics13030231 (PMC10967342; doi:10.3390/antibiotics13030231)
Supplement: Supplementary file 1 [file antibiotics-13-00231-s001.zip › Supplementary File S1.zip!/Supplementary File S1/MAPt Raw Data/Supplementary 4 readme.pdf]

Supplementary 4:

Samples 1-17:

Column A: The well position in the PCR.

Column B: Antibiotic treatment

Column C: Antibiotic concentrations.

Column D: Raw Ct value.

Column E: ΔCT between a treated sample and the first growth control (GC).

Column F: ΔCT between a treated sample and the second growth control (GC).

Column G: The average between column E and F.

Samples 18-63:

Column A: The well position in the PCR.

Column B: Raw Ct value.

Row named Ct: The raw Ct from column B according to their antibiotic name and concentration.

Row named Ct-average G.C.: The ΔCT between a treated sample and the growth controls average (GC).
